# The effects of facial expressions on judgments of others when observing two-person confrontation scenes from a third person perspective

**DOI:** 10.3389/fpsyg.2022.856336

**Published:** 2022-09-27

**Authors:** Yoshiyuki Ueda, Sakiko Yoshikawa

**Affiliations:** ^1^Institute for the Future of Human Society, Kyoto University, Kyoto, Japan; ^2^Faculty of Art and Design, Kyoto University of the Arts, Kyoto, Japan

**Keywords:** social interaction, facial expression, intuitive judgment, deliberative judgment, personality traits

## Abstract

When building personal relationships, it is important to select optimal partners, even based on the first meeting. This study was inspired by the idea that people who smile are considered more trustworthy and attractive. However, this may not always be true in daily life. Previous studies have used a relatively simple method of judging others by presenting a photograph of one person’s face. To move beyond this approach and examine more complex situations, we presented the faces of two people confronted with each other to participants and asked them to judge them from a third-person perspective. Through three experiments, participants were asked to judge which of the two persons was more appropriate for forming alliances, more trustworthy, or more attractive, respectively. In all experiments, images were shown for a short (500 ms) or a long time (5 s). In all three experiments, the results showed that participants were more likely to choose persons with happy faces than those with neutral, sad, or angry faces when the image presentation was short. Contrarily, the facial expressions did not affect those judgments when the image presentation was long. Instead, judgments were correlated with personality estimated from the model’s neutral face in a single-person presentation. These results suggest that although facial expressions can affect the judgments of others when observing two-person confrontations from a third-person perspective, when participants have more time to elaborate their judgments, they go beyond expressions.

## Introduction

Throughout our lifetime, we meet many individuals and build relationships with them. The question is: how do we assess other people’s personalities to search for someone to build a relationship with? An assumption often used is that the personality of an individual can be inferred using appearance (i.e., facial identity and facial expressions) as a cue. Accordingly, many studies have examined how personality was inferred from facial appearances by presenting a single facial expression (e.g., Knutson, 1996; Hassin and Trope, 2000; Willis and Todorov, 2006; Ballew and Todorov, 2007; Todorov et al., 2008; Naumann et al., 2009; Little and Perrett, 2010; Olivola et al., 2014; Hehman et al., 2015). Their results demonstrated that happy expressions often convey a sign of trustworthiness, whereas angry expressions signal hostility, aggression, and physical dominance (Keating et al., 1981; Knutson, 1996; Hall et al., 2005; Todorov et al., 2008). More interestingly, impressions can be formed within 100 ms, and they do not change greatly even when the presentation time increases (Willis and Todorov, 2006). In other words, people tend to overgeneralize their judgments at first glance (Zebrowitz and Montepare, 2008).

Although many of these studies have been examined in situations where people are directly facing the other person, human communication and social interaction styles are not limited to face-to-face interactions. If what happens in a real social environment is to be known, it may be too simplistic to infer personality traits by looking at one person’s face from a direct face-to-face perspective, as has been done in previous studies. For this reason, recent studies have observed the interaction of others from a third-person perspective, a position that is one step back (Ueda and Yoshikawa, 2018; Vestner et al., 2019, 2021; Abassi and Papeo, 2020). In this framework, Ueda and Yoshikawa (2018) showed that the dominant personality is inferred when facing one person (hereafter referred to as the single-person situation), but participants did not predict which one was most dominant when watching the interaction between two people (referred to as the third-person perspective or confrontation situation). This indicates that personality traits that have been examined in the first-person situation need to be re-examined to see if they work in the third-person perspective situation.

Moreover, Vestner et al. (2021) assumed two forms of processing in the social binding theory, in which differences in perception between the single-person situations and the third-person perspective situations were explained. The initial process is automatic, representing the interacting individuals as a single entity, whereas the following process separately represents the characteristics attributed to each individual. Considering these two processes, the personality impression inferred in a third-person perspective situation may differ between short and long presentation durations. Shorter presentation durations rely more on the first automatic component, representing the interacting individuals as a single entity, whereas longer presentation durations drive the other process of focusing attention on each individual and separately representing each individual’s characteristics as associated with that person, which influences the decision. In the heuristic-systematic model of information processing (Chaiken, 1980; Chaiken and Ledgerwood, 2012), people judge heuristically when they have less knowledge, time, and motivation. Whereas systematic processing is likely to occur when they can devote enough attention and would provide support for this prediction.

Contrary to this expectation, however, Ueda and Yoshikawa (2018) showed that people with happy expressions were always judged as more dominant in groups, both in shorter exposure duration (i.e., 200 ms) and longer exposure duration (e.g., 10 s). Since dominance is situation-dependent, i.e., judgments that are weighted toward the current situation and relationships among individuals (Ueda et al., 2017), it may always be strongly dependent on signals of the current state, such as facial expressions. Although trustworthiness and dominance are considered the two main orthogonal dimensions of face evaluation (Oosterhof and Todorov, 2008), the previous study examined only the dominance dimension from the third-person perspective. Some previous studies examined trustworthiness and attractiveness in single-person situations (Oosterhof and Todorov, 2008; Sutherland et al., 2018), and impressions do not change greatly depending on the presentation time (Abassi and Papeo, 2020), but no study has examined the situations in which two persons face each other. Therefore, we cannot say for sure that a face expressing happiness is always trustworthy or attractive in every situation. Some theories, such as social binding and heuristic-systematic models, allow us to predict that the effects of facial expressions will vary with presentation time. To shed light on social cognition in a more general context, in this study, we investigated the mechanisms that judge the dimension of trustworthiness in the third-person perspective and its temporal consistency.

This study aims to investigate the role of facial expressions when people judge others on the dimension of trustworthiness in confrontational situations using two presentation durations (i.e., short or long). To achieve this goal, we employed the same method used by Ueda and Yoshikawa (2018): participants were presented with a scene of two people facing each other and then judged which person was more dominant. Since Todorov and Portner (2014) suggest that the context under which social judgments are made is important, we asked participants to evaluate the people in the group from three perspectives: Who was more appropriate for socializing and forming alliances (Experiment 1), Who was more trustworthy (Experiment 2), and Who was more attractive (Experiment 3). Furthermore, we compared personality trait ratings inferred in single-person situations with the results obtained in confrontation situations.

In single-person situations, the dimension of trustworthiness was strongly correlated with the discrimination of happiness and anger expressions: the happier the emotion a face expresses, the more trustworthy those faces are perceived (Oosterhof and Todorov, 2008). Additionally, a smile evoked the impression that a person was trustworthy and extraverted (Todorov and Portner, 2014). Moreover, participants perceived those with baby-like and feminine faces to be more trustworthy than those with mature and masculine faces (Oosterhof and Todorov, 2008). Therefore, if the observations of single-person situations could be directly extended to the confrontation situations, we would expect that people with these characteristics are more likely to be selected as desirable individuals. However, since the effect of presentation duration in confrontation situations is unknown, we do not know whether the same effect is found when the presentation is instantaneous (i.e., required intuitive judgments) or when it is prolonged (i.e., allowing deliberative judgments).

In addition to temporal consistency, we also considered another possible effective factor concerning social perception in confrontation situations: the intensity of facial expressions. The perceived intensity of expressions varied linearly in accordance with their physical intensity (Hess et al., 1997). However, it remains unclear whether personality signals conveyed *via* facial expressions varied in the same way in accordance with the physical intensity of facial expressions in confrontation situations. If the degree of expressiveness of facial expressions can lead to different interpretations of the kind of person he/she is, then the person selected may differ depending on how strongly they express his/her expressions. Conversely, if it is consistently interpreted despite changes in intensity, then consistent results may be obtained across different intensity levels of facial expressions.

It should be noted that complicated social interactions are not limited to situations in which an observer sees two persons interacting, but this study is the first step in examining the effect of different social interactions on perceived trustworthiness and attractiveness. Furthermore, by using this presentation situation, we can compare the results of this study with the results of the dominance dimension of the previous study (Ueda and Yoshikawa, 2018).

## Materials and methods

The study consisted of three distinct experiments. The design of each study was as follows. In Experiment 1, we examined whom among the two people was judged as more appropriate for socializing or forming alliances and how facial expression influenced this decision. This experiment was conducted by manipulating the duration of the face presentation and the physical intensity of expressions. In Experiments 2 and 3, we examined trustworthiness and attractiveness judgments using the same methods as Experiment 1. Since some personality traits, such as trustworthiness, have been reported to be associated with feminine/masculine facial features (Oosterhof and Todorov, 2008), presenting men and women in pairs, or looking at both male and female pairs could have a carryover effect. Therefore, we divided the experiments into two parts as in the previous study (Ueda and Yoshikawa, 2018): one in which participants were presented with male pairs and the other one in which they were presented with female pairs. Finally, we investigated the effect of perceived personality traits inferred from facial identities in single-person situations on judgments in confrontation situations. This research was conducted according to the principles expressed in the Declaration of Helsinki. In all experiments, we report all measures, manipulations, and exclusions.

### Informed consent and sample size

Informed consent (including the study’s purpose, methodology, risks, the right to withdraw, duration of the experiment, handling of individual information, and voluntary nature of participation) was provided by all participants prior to the experiment. All participants had normal or corrected-to-normal vision and were naïve concerning the purpose of the experiment.

Before Experiment 1, the sample size was determined to be 20 for men and women pairs each, based on a calculation of the required sample size with *α* = 0.05, 1–*β* = 0.80, and effect size *d* = 0.59, which was based on the previous study (Ueda and Yoshikawa, 2018). It was then applied throughout the experiments in this study.

### Materials

#### Apparatus

The experiment was conducted individually in a soundproof chamber. MATLAB (MathWorks) with Psychophysics Toolbox extension (Brainard, 1997; Pelli, 1997; http://psychtoolbox.org/) controlled stimuli presentation on a CRT monitor (Dell UltraScan P991, 19 inches, 35 cm × 26 cm) with a resolution of 1,024 × 768 pixels and a refresh rate of 60 Hz. The distance between the participant’s head and monitor was fixed at 75 cm using a chinrest.

#### Stimuli

Forty-eight photos of 12 women with four facial expressions (happiness, anger, sadness, and neutral) and 48 photos of 12 men with four facial expressions were used. The photos were selected from the Kokoro Research Center’s (KRC) facial expression database (Ueda et al., 2019). Each photo model expressed all four facial expressions used in this experiment. Twenty-eight participants, including 14 females, were asked to rate each photo in terms of how strongly the emotions of happiness, anger, and sadness the model in the photo expressed on a 7-point scale (1 = *very weak*, 7 = *very strong*). The results showed that happy (4.65 ± 0.67), angry (4.38 ± 0.43), and sad (4.58 ± 0.62) expressions were recognized easily, and the recognizabilities were almost identical. Contrarily, neutral expressions were rated as much less intense (all expressions were < 2.35).

We manipulated the intensity of facial expressions. The intensity of a facial expression means how strongly an emotion is perceived from that expression. Since this correlates with how greatly the facial muscles moved from neutral facial expressions, morphing was used to manipulate how much the facial expression changed from neutral expressions in the experiment. We morphed faces with happy, angry, or sad expressions with neutral expressions and created facial expressions with 50% intensity (using FantaMoroh provided by Abrosoft). The intensity of 100% refers to a full happy, angry, or sad face (i.e., original images in the database), 0% refers to a neutral expression, and 50% refers to a face morphed 50/50 between happy, angry, or sad and neutral expressions.

Each photo faced either left or right, and opposite orientations were created with mirror images. The visual angle subtended by each picture was 13.6° × 15.6°.

### Experiment 1: Whose side do people want to be on?

#### Participants

Forty graduate and undergraduate students from Kyoto University, including 18 women, participated. Their ages ranged from 19 to 31 years (*M* = 21.6, *SD* = 2.2). Due to the time constraints of the experiment, only female faces were presented to half the participants (a group comprised of both sexes), and only male faces were presented to the other half.

#### Procedure

**Figure 1:**
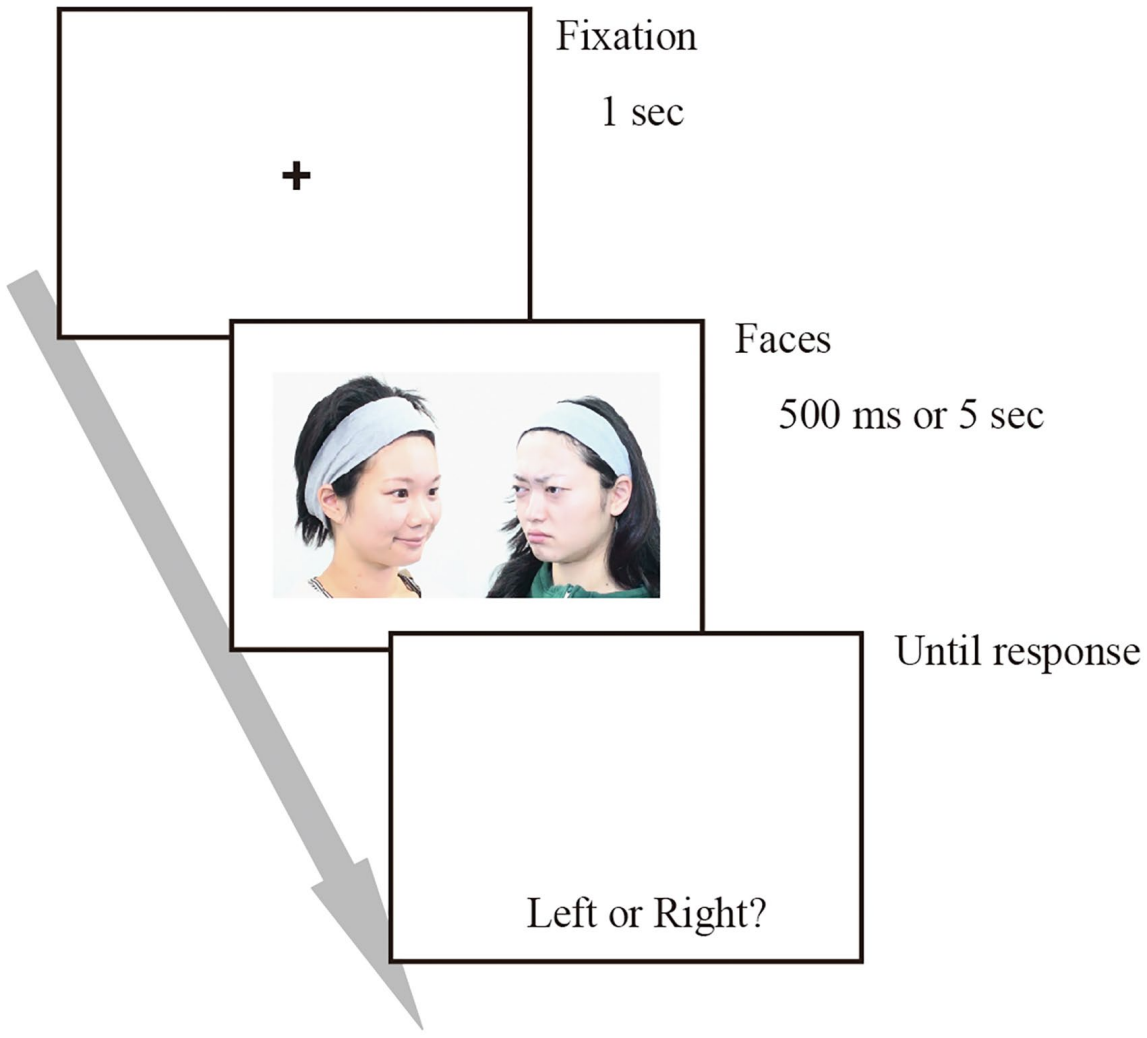
Schema for the experiments. Two individuals were facing each other.

At the beginning of each trial, a fixation cross was presented at the centre of the monitor, accompanied by a short beep. After 1 s, two photos were presented on the left and right sides of the monitor (approximately 6.8° visual angle from the centre of the monitor; see Figure 1). All possible pairs of facial expressions were presented. In the strong expression trials, original (intact) facial expression faces were presented, whereas, in the weak expression trials, facial expressions with 50% intensity were presented. In every trial, different photo models were randomly selected and assigned to each expression. The photos presented on the left side had the model facing to the right, whereas the photos presented on the right side had the model facing to the left, creating two persons facing each other.

Participants were asked to assume that the two people were interacting socially and to judge which person’s side they wanted to be on. Images were presented for either 500 ms or 5 s, depending on the session. After the presentation, the photos disappeared, and participants reported their judgments. Although no time limit was set for responses, participants were instructed to answer as they felt and not to take time to overthink their answers.

The experiment was divided into two equal sessions, each involving a different presentation duration condition, for a total of 288 trials: two sessions (one for each presentation duration) with four blocks per session and 36 trials per block. There were 12 repetitions of each possible facial-expression pair at each intensity level (100 and 50% intensity) and in each presentation duration condition. The presentation location of each expression among each pair was counterbalanced within a participant. The presentation order for each pair was random, and the session order (presentation duration) was counterbalanced across participants. Before the experiment, participants performed two practice trials to understand the trial procedure, but responses were not recorded in the practice trials.

#### Analysis

As response tendencies were the same in trials with 100 and 50% intensity facial expression photos, those trials were combined for analysis. Also, since response tendencies were the same across participants who were presented with only female faces and only male faces, they were combined.

Participants chose one of the pairs during each trial. This method is referred to as two-alternative forced-choice (2AFC), which allows us to detect even slight differences through repeated measurements.

In the first analysis, we examined whether the probability of choosing one expression was different from the chance level (=50%) when it was paired with another expression. There were 24 repetitions for each pair of expressions. Therefore, we conducted a *t*-test using the average selection rate of each participant’s 24 trials as the dependent variable to examine if it differed from the chance level. Moreover, a one-way ANOVA with six levels (i.e., pairs with happiness and neutral, happiness and sadness, happiness and anger, neutral and sadness, neutral and anger, and sadness and anger) of reaction times (RT) was conducted to examine whether there were differences between the pairs.

Although the results of the *t*-tests only show which of the expressions in each pair was significantly selected, it cannot examine which of the four facial expressions was the most selected. To integrate the results of each pair and examine which expressions were the most selected, in the second analysis, we applied Nakaya’s variation of Scheffé’s pairwise comparison method (Nakaya, 1970; Scheffé, 1952). In this method, all pairs are compared in multiple grades (for example, participants grade the difference between stimulus A and stimulus B on a 5-point scale). Since 2AFC was repeated 24 times for each pair in this experiment, the number of choices among the 24 repetitions was used as the grade. Here, the grade was set to 0 when two facial expressions in a pair were equally selected and calculated as [the number of selections – 12]. For example, if one participant selected happy expressions 22 times among 24 repetitions of a pair of happy and neutral expressions, the grades of happiness and neutral were 10 (= 22–12) and-10 (= 2–12), respectively, for that pair. Facial expressions of all pairs were then represented from 12 (= 24–12) to-12 (= 0–12). We represented the average grade for each facial expression across pairs and participants on a scale and conducted a one-way ANOVA with four levels (i.e., happiness, neutral, sadness, and anger) on an average grade to examine the differences among four facial expressions.

These analyses were common across experiments.

### Experiment 2: Who is more trustworthy?

#### Participants

Forty graduate and undergraduate students from Kyoto University, including 20 women, participated. Their ages ranged from 18 to 25 years (*M* = 21.1, *SD* = 1.9). As in Experiment 1, only female faces were presented to half of the participants (a group comprised of both sexes), and only male faces were presented to the other half.

#### Procedure

In this experiment, we asked participants to assume two people were socially interacting and to judge Who was more trustworthy. Other procedures were identical to Experiment 1.

### Experiment 3: Who is more attractive?

#### Participants

Forty graduate and undergraduate students from Kyoto University, including 19 women, participated. Their ages ranged from 18 to 34 years (*M* = 20.8, *SD* = 2.8). As in Experiments 1 and 2, only female faces were presented to half of the participants (a group comprised of both sexes), and only male faces were presented to the other half.

#### Procedure

In this experiment, we asked participants to assume two people were socially interacting and judge Who was more attractive. Other procedures were identical to Experiment 1.

## Results and discussion

### Experiment 1: Which person’s side do people want to be on?

#### Results

For each participant, the ratios of those judged more desirable to be with were calculated, and then they were averaged for each presentation pair. The average ratios are shown in Table 1 for the 500 ms presentation duration condition (hereafter 500 ms condition) and Table 2 for the 5 s presentation duration condition (hereafter 5 s condition). The results of RTs are shown in Table 3.

**Table 1:** Average ratio of chosen emotions in 500 ms presentation of Experiment 1.

	Compared with			
	Happy	Neutral	Sad	Angry
Happy		0.66	0.78	0.78
Neutral	0.34		0.71	0.73
Sad	0.22	0.29		0.54
Angry	0.22	0.27	0.46	

Each cell indicates a ratio showing that the expression depicted on the lower left side was chosen more often than the emotion depicted on the upper right side when they were paired. For example, a happy expression was chosen 72% of the time and neutral expression was chosen 28% of the time when happy and neutral expressions were confronted. The same is true in the following Tables 2, 4, 5, 6, and 7.

**Table 2:** Average ratio of chosen emotions in 5,000 ms presentation of Experiment 1.

	Compared with			
	Happy	Neutral	Sad	Angry
Happy		0.52	0.51	0.50
Neutral	0.48		0.51	0.51
Sad	0.49	0.49		0.50
Angry	0.50	0.49	0.50	

**Table 3:** Average reaction times for each facial expression pair.

		Happy vs. Neutral		Happy vs. Sad		Happy vs. Angry		Neutral vs. Sad		Neutral vs. Angry		Sad vs. Angry	
Experiment 1	500 ms	588	(108)	585	(83)	592	(100)	595	(103)	612	(119)	655	(169)
	5 s	694	(223)	692	(222)	719	(300)	684	(198)	688	(200)	706	(223)

Experiment 2	500 ms	739	(260)	735	(277)	715	(254)	725	(226)	738	(246)	790	(304)
	5 s	754	(333)	740	(257)	728	(239)	756	(385)	745	(322)	753	(303)

Experiment 3	500 ms	668	(300)	664	(274)	666	(299)	715	(314)	680	(256)	787	(353)
	5 s	680	(276)	674	(270)	673	(325)	720	(366)	697	(308)	700	(255)

The units for each cell are milliseconds. The numbers in the parentheses indicate the standard deviation.

First, we conducted *t*-tests to examine whether one facial expression was selected significantly more often than chance level. Since there are six pairs, the significance level was set to 0.0083 (= 0.05/6). In the 500 ms condition, people with happy facial expressions were judged significantly more desirable to be with than people with other expressions [compared with neutral, *t*(39) = 6.24, *p* = 2.43 × 10^−7^, *d* = 0.99, angry, *t*(39) = 10.29, *p* = 1.12 × 10^−12^, *d* = 1.63, and sad, *t*(39) = 9.93, *p* = 3.16 × 10^−12^, *d* = 1.57]. Moreover, people with neutral facial expressions were judged significantly more desirable to be with than those with angry and sad expressions [*t*(39) = 10.27, *p* = 1.20 × 10^−12^, *d* = 1.62 and *t*(39) = 10.19, *p* = 1.19 × 10^−12^, *d* = 1.61, respectively], while ratio was not different between people with angry and sad expressions, *t*(39) = 1.62, *p* = 0.11, *d* = 0.26.

On the other hand, in the 5 s condition, the results demonstrated no significant differences in selection ratios among facial expressions [happy vs. neutral, *t*(39) = 1.47, *p* = 0.15, *d* = 0.23; happy vs. angry, *t*(39) = 0.06, *p* = 0.95, *d* = 0.01; happy vs. sad, *t*(39) = 0.83, *p* = 0.41, *d* = 0.13; neutral vs. angry, *t*(39) = 0.75, *p* = 0.46, *d* = 0.12; neutral vs. sad, *t*(39) = 0.50, *p* = 0.62, *d* = 0.08; angry vs. sad, *t*(39) = 0.14, *p* = 0.89, *d* = 0.02]. These results suggested that judgments made during a longer presentation duration were not explained solely by the models’ facial expressions.

For the analyses of RTs, we excluded one participant whose RT was larger than the mean + 3*SD* of all participants. In the 500 ms condition, an ANOVA with six facial expression pairs demonstrated a main effect of pairs, *F*(5, 190) = 7.15, *p* = 3.73 × 10^−6^, $\eta_{P}^{2}$ = 0.16. Multiple comparisons using Shaffer’s modified sequentially rejective Bonferroni procedure showed that participants took longer time for pairs with angry and sad expressions than others, *p_adj_* < 0.035 (however, the differences between pairs with angry and sad and those with happy and sad expressions were marginally significant, *p_adj_* = 0.073). The results suggest that the response time was slow in pairs, where the selection rate did not differ from the chance level.

In the 5 s condition, there was no significant main effect of pairs on RT, *F*(5, 190) = 0.43, *p* = 0.83, $\eta_{P}^{2}$ = 0.01.

**Figure 2:**
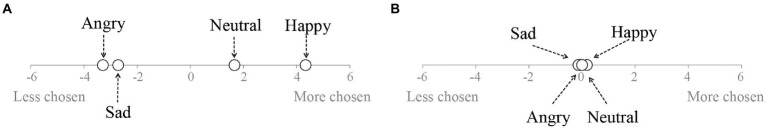
Results of experiment 1: Who is the best to be with? Mean of the grade on the scale in partner judgments with 500 ms presentation **(A)** and 5 s presentation **(B)**.

Second, we quantified which facial expression was selected more often. The average grade for each facial expression is shown in Figures 2A,B for the 500 ms and 5 s conditions, respectively. For the 500 ms condition, one-way ANOVA with four-level of facial expressions demonstrated a main effect, *F*(3, 117) = 458.81, *p* ≈ 0, $\eta_{P}^{2}$ = 0.92. Multiple comparisons based on the 95% confidence interval demonstrated that people with happy facial expressions were judged significantly more desirable to side with than those with other expressions, and people with neutral facial expressions were selected more than those with angry and sad expressions. However, there was no significant difference in the selection ratio between people with angry or sad expressions.

For the 5 s condition, an ANOVA demonstrated no main effect of facial expressions, *F*(3, 117) = 0.47, *p* = 0.70, $\eta_{P}^{2}$ = 0.01. The result indicated that facial expressions could not explain the judgments in longer, deliberative consideration.

Exploratory, to examine the effects of model sex and participant sex on results, we conducted the second analysis again, separately, for model sex and participant sex. Each scale is shown in Supplementary Table S1. When model sex and participant sex were women/women (*n* = 9), women/men (*n* = 11), and men/women (*n* = 9), the results were identical to the overall results. When both model sex and participant sex were men (*n* = 11), neutral expressions had a larger average grade than happy expressions in the 500 ms condition, and no statistically significant differences between neutral and happy expressions were found. In this case, although people with happy expressions were chosen more than those with neutral expressions when people with happy and neutral expressions were paired, people with neutral expressions were chosen more than those with happy expressions when they were paired with other expressions (i.e., angry and sad expressions). We should note that this analysis has only a small number of participants and is exploratory, but the difference between neutral and happy expressions may be smaller when men are making judgments about men.

#### Discussion

In experiment 1, individuals with happy expressions were selected as more desirable to side with based on instantaneous or intuitive judgments (i.e., 500 ms condition) in confrontation situations. Since faces with happy expressions convey trustworthiness/attractiveness (Oosterhof and Todorov, 2008; Golle et al., 2014; specifically, for women, Tracy and Beall, 2011), participants may have selected individuals based on a trustworthiness and attractiveness component. On the other hand, this effect disappeared with deliberative consideration (i.e., 5 s presentation duration). This finding was inconsistent with single-person situations of previous research (Willis and Todorov, 2006; Ueda and Yoshikawa, 2018), demonstrating that personality traits inferred in short-exposure durations were highly correlated with those inferred in unconstrained exposure durations. In experiments with single-person situations, observers had no cues to infer personality traits except for facial expressions, and therefore, they used to evaluate personality traits based on facial expressions regardless of presentation durations. Compared with them, in this study, although for the shorter presentation condition, participants depended on facial expressions as strong cues in confrontation situations, given a longer time (i.e., 5 s condition), participants could have compared the two faces and used other cues (e.g., personality estimated from the faces).

### Experiment 2: Who is more trustworthy?

#### Results

For each paired facial expression, the average ratios of trustworthiness judgments are shown in Table 4 for the 500 ms condition and Table 5 for the 5 s conditions. The results of RTs are shown in Table 3.

**Table 4:** Average ratio of chosen emotions in 500 ms presentation of Experiment 2.

	Compared with			
	Happy	Neutral	Sad	Angry
Happy		0.59	0.72	0.69
Neutral	0.41		0.70	0.68
Sad	0.28	0.30		0.49
Angry	0.31	0.32	0.51	

**Table 5:** Average ratio of chosen emotions in 5,000 ms presentation of Experiment 2.

	Compared with			
	Happy	Neutral	Sad	Angry
Happy		0.49	0.53	0.51
Neutral	0.51		0.51	0.49
Sad	0.47	0.49		0.52
Angry	0.49	0.51	0.48	

*T*-tests comparing chance level demonstrated that, in the 500 ms condition, people with happy facial expressions were more trustworthy than those with angry and sad expressions [angry, *t*(39) = 4.92, *p* = 1.61 × 10^−5^, *d* = 0.78, and sad, *t*(39) = 6.18, *p* = 2.90 × 10^−7^, d = 0.98, respectively]. They were also more trustworthy than those with neutral expressions, but did not reach the significant level, *t*(39) = 2.40, *p* = 0.021, *d* = 0.38. Furthermore, people with neutral expressions were more trustworthy than those with angry and sad expressions [*t*(39) = 6.04, *p* = 4.50 × 10^−7^, *d* = 0.96, and *t*(39) = 6.42, *p* = 1.36 × 10^−7^, *d* = 1.01, respectively]. However, the selection ratio was not different between people with angry and sad expressions, *t*(39) = 0.47, *p* = 0.64, *d* = 0.07.

On the other hand, in the 5 s condition, the results demonstrated no significant differences in selection ratios among facial expressions [happy vs. neutral, *t*(39) = 0.34, *p* = 0.73, *d* = 0.05; happy vs. angry, *t*(39) = 0.29, *p* = 0.77, *d* = 0.05; happy vs. sad, *t*(39) = 2.00, *p* = 0.05, *d* = 0.32; neutral vs. angry, *t*(39) = 0.89, *p* = 0.38, *d* = 0.14; neutral vs. sad, *t*(39) = 0.82, *p* = 0.42, *d* = 0.13; and angry vs. sad, *t*(39) = 1.36, *p* = 0.18, *d* = 0.22].

For the analyses of RTs, we excluded one participant whose RT was larger than the mean + 3*SD* of all participants. In the 500 ms condition, an ANOVA with six facial expression pairs demonstrated a main effect of pairs, *F*(5, 190) = 2.96, *p* = 0.013, $\eta_{P}^{2}$ = 0.07, but multiple comparisons using Shaffer’s modified sequentially rejective Bonferroni procedure showed no differences between any pairs, *p_adj_* > 0.21. In the 5 s condition, there was no significant main effect of pairs, *F*(5, 190) = 0.26, *p* = 0.93, $\eta_{P}^{2}$ = 0.01.

**Figure 3:**
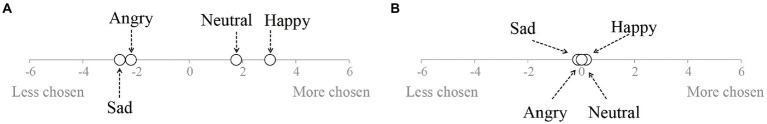
Results of experiment 2: Who is more trustworthy? Mean of the grade on the scale in trustworthiness judgments with 500 ms presentation **(A)** and 5 s presentation **(B)**.

The averages of the trustworthiness grades of facial expressions on the scale are shown in Figures 3A,B. For the 500 ms condition, an ANOVA demonstrated a main effect of facial expressions, *F*(3, 117) = 199.76, *p* ≈ 0, $\eta_{P}^{2}$ = 0.84. Multiple comparisons based on a 95% confidence interval demonstrated that people with happy facial expressions were judged more trustworthy than those with other expressions, and people with neutral facial expressions were selected more than those with angry and sad expressions. However, there was no significant difference in the selection ratio between people with angry expressions and sad expressions.

For the 5 s condition, an ANOVA demonstrated no main effect of facial expressions, *F*(3, 117) = 0.43, *p* = 0.73, $\eta_{P}^{2}$ = 0.01.

Exploratory analysis to separate the effect of model sex and participant sex (see Supplementary Table S2) showed that when both were women/women (*n* = 10) and men/men (*n* = 10), the results were identical to the overall results. When model sex and participant sex were women/men (*n* = 10) and men/women (*n* = 10), there were no statistically significant differences between happy and neutral expressions even in the 500 ms condition.

#### Discussion

In experiment 2, the results indicated that the effect of facial expressions on trustworthiness judgment was the same as those in Experiment 1: instantaneous judgments were strongly affected by facial expressions, but facial expressions did not explain judgments with a longer duration in confrontation scenes. The results of Experiment 2 did not support previous results that perceived trustworthiness strongly correlated regardless of presentation durations in single-person situations (Willis and Todorov, 2006). Although people with happy facial expressions were chosen as more trustworthy than those with neutral expressions in the 500 ms condition, this effect was not as strong as in Experiment 1.

### Experiment 3: Who is more attractive?

#### Results

For each paired facial expression, the average ratios of attractiveness judgments are shown in Table 6 for the 500 ms condition, and Table 7 for the 5 s condition. The results of RTs are shown in Table 3.

**Table 6:** Average ratio of chosen emotions in 500 ms presentation of Experiment 3.

	Compared with			
	Happy	Neutral	Sad	Angry
Happy		0.70	0.79	0.79
Neutral	0.30		0.70	0.70
Sad	0.21	0.30		0.49
Angry	0.21	0.30	0.51	

**Table 7:** Average ratio of chosen emotions in 5,000 ms presentation of Experiment 3.

	Compared with			
	Happy	Neutral	Sad	Angry
Happy		0.54	0.51	0.53
Neutral	0.46		0.50	0.53
Sad	0.49	0.50		0.54
Angry	0.47	0.47	0.46	

*T*-tests comparing with the chance level demonstrated that, in the 500 ms condition, people with happy facial expressions were more attractive than those with other expressions [compared with neutral, *t*(39) = 6.15, *p* = 3.16 × 10^−7^, *d* = 0.97, angry, *t*(39) = 9.06, *p* = 3.84 × 10^−11^, *d* = 1.43, and sad, *t*(39) = 10.14, *p* = 1.73 × 10^−12^, *d* = 1.60]. Furthermore, people with neutral expressions were more attractive than those with angry or sad expressions [*t*(39) = 7.54, *p* = 3.95 × 10^−9^, *d* = 1.19, and *t*(39) = 7.67, *p* = 2.60 × 10^−9^, *d* = 1.21, respectively]. However, selection ratio was not different between people with angry and sad expressions, *t*(39) = 0.52, *p* = 0.61, *d* = 0.08.

On the other hand, in the 5 s condition, although selection ratio of people with happy expressions was marginally higher than those with neutral expressions, *t*(39) = 2.88, *p* = 0.006, *d* = 0.46, other results demonstrated no significant differences among facial expressions [happy vs. angry, *t*(39) = 2.03, *p* = 0.05, *d* = 0.32; happy vs. sad, *t*(39) = 0.41, *p* = 0.68, d = 0.06; neutral vs. angry, *t*(39) = 1.88, *p* = 0.07, *d* = 0.30; neutral vs. sad, *t*(39) = 0.29, *p* = 0.77, *d* = 0.05; and angry vs. sad, *t*(39) = 2.09, *p* = 0.04, *d* = 0.33].

For the analyses of RTs, we excluded three participants whose RTs were larger than the mean + 3*SD* of all participants. In the 500 ms condition, an ANOVA with six facial expression pairs demonstrated a main effect of pairs, *F*(5, 180) = 9.29, *p* = 6.93 × 10^−8^, $\eta_{P}^{2}$ = 0.21. Multiple comparisons using Shaffer’s modified sequentially rejective Bonferroni procedure showed that participants took longer time for pairs with angry and sad expressions than others, *p_adj_* < 0.040, and longer for pairs with neutral and sad expressions than those with happy and neutral expressions, *p_adj_* = 0.047, and those with happy and sad expressions, *p_adj_* = 0.047.

In the 5 s condition, there was no significant main effect of pairs on RT, *F*(5, 180) = 0.93, *p* = 0.46, $\eta_{P}^{2}$ = 0.03.

**Figure 4:**
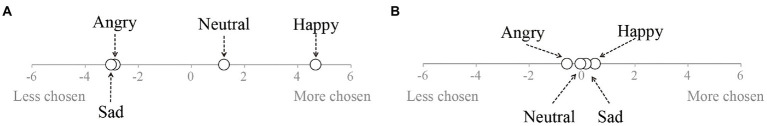
Results of experiment 3: Who is more attractive? Mean of the grade on the scale in attractiveness judgments with 500 ms presentation **(A)** and 5 s presentation **(B)**.

The averages of the attractiveness grades of facial expressions on the scale are shown in Figures 4A,B. For the 500 ms condition, an ANOVA demonstrated a main effect of facial expressions, *F*(3, 117) = 350.92, *p* ≈ 0, $\eta_{P}^{2}$ = 0.90. Multiple comparisons based on the 95% confidence interval demonstrated that people with happy facial expressions were judged more attractive than those with other expressions, and people with neutral facial expressions were selected more than those with angry or sad expressions. However, there was no significant difference in the selection ratio between people with angry expressions and sad expressions.

For the 5 s condition, an ANOVA demonstrated a main effect of facial expressions, *F*(3, 117) = 4.93, *p* = 0.003, $\eta_{P}^{2}$ = 0.11. Multiple comparisons based on a 95% confidence interval demonstrated that people with happy facial expressions were selected as more attractive than those with angry expressions.

Exploratory analysis to separate the effect of model sex and participant sex (see Supplementary Table S3) showed that each result was identical to the overall results.

#### Discussion

Experiment 3 showed that instantaneous attractiveness judgments were strongly affected by models’ facial expressions. In deliberative judgments with longer exposure duration, although participants selected people with happy expressions as more attractive persons, this effect was much smaller than in the instantaneous judgment. These results were generally consistent with Experiments 1 and 2, suggesting that for attractiveness judgments, participants used different cues between instantaneous responses and deliberative responses in confrontation situations.

### *Ad-hoc* analyses: Who was selected more often in deliberative judgments?

In deliberative judgments (i.e., judgments of 5 s exposure duration), the facial expressions could not fully explain which individuals were selected more often. To reveal what factors participants used to make this judgment, we conducted *Ad-hoc* analyses in which we investigated correlations between the selection ratio of each model in deliberative judgments and their personality traits estimated in single-person situations. For personality traits of photo models estimated in single-person situations, we used face evaluation values recorded in the KRC facial expression database, i.e., attractiveness, compassion, competence, distinctiveness, dominance, extraversion, maturity, and trustworthiness (Ueda et al., 2019). These values were obtained from the ratings of 31 participants who were different people that participated in this study when they viewed each face with neutral expressions.

The correlations are shown in Table 8. The results suggested that selection ratios in deliberative judgments were strongly correlated with some of the estimated personality traits of photo models. Specifically, for overall models (i.e., including both female and male faces) attractiveness, compassion, competence, extraversion, and trustworthiness were strongly correlated. The principal component analysis in Ueda et al. (2019) demonstrated that these personality traits contribute to the first principal component of face evaluation, i.e., the trustworthiness dimension, whereas distinctiveness, dominance, and maturity contributed to the second principal component of face evaluation, i.e., the dominance dimension. This suggested that for judgements of social (or alliance) partner selection, trustworthiness, and attractiveness in confrontation situations, participants relied primarily on the trustworthiness dimension alone when they could deliberate.

**Table 8:** Correlations between choice ratios in deliberative judgments and estimated personality traits.

		Attractiveness	Compassion	Competence	Distinctiveness	Dominance	Extraversion	Maturity	Trustworthiness
Overall Model (*n* = 24)	Exp. 1	0.75	0.61	0.69	0.15	0.08	0.51	−0.31	0.67
	Exp. 2	0.54	0.50	0.51	0.30	0.15	0.45	−0.02	0.52
	Exp. 3	0.73	0.59	0.70	0.28	0.21	0.61	−0.15	0.64

Female Model (*n* = 12)	Exp. 1	0.70	0.71	0.62	−0.11	−0.32	0.18	−0.69	0.72
	Exp. 2	0.69	0.74	0.55	−0.03	−0.31	0.19	−0.53	0.77
	Exp. 3	0.81	0.75	0.73	0.14	−0.13	0.39	−0.47	0.77

Male Model (*n* = 12)	Exp. 1	0.79	0.56	0.76	0.37	0.57	0.83	−0.03	0.65
	Exp. 2	0.46	0.37	0.50	0.57	0.61	0.69	0.32	0.39
	Exp. 3	0.67	0.48	0.70	0.43	0.70	0.87	0.15	0.55

Grey cells indicate that correlation coefficients were significantly different from zero (two-tailed: *p* < 0.05).

Interestingly, correlation patterns differed between female and male models. In female-face pairs, attractiveness, compassion, competence, and trustworthiness were strongly correlated with selection ratios. That is, the more attractive, compassionate, competent, and trustworthy the woman appeared to be, the more often she was regarded as the most desirable person to side with as well as attractive and trustworthy. However, for male-face pairs, compassion and trustworthiness were not strongly correlated; instead, dominance and extraversion were correlated with the selection ratios. This suggested that participants used different personality traits as cues according to the model’s sex. Specifically, they did not rely on the trustworthiness dimension alone but also on the dominance dimension for male-face pairs.

The correlation coefficients observed in Experiment 1 were closer to those observed in Experiment 3 rather those in Experiment 2. This indicated that social (or alliance) partner judgments may be weighted more towards the model’s attractiveness rather than trustworthiness.

## General discussion

During instantaneous or intuitive judgments (i.e., shorter exposure duration), participants made judgments based on facial expressions. Models that expressed happiness were selected much more often than those that expressed other emotions. However, in deliberative judgments (i.e., longer exposure duration), participants were no longer affected by models’ facial expressions. We calculated the selection ratios of each expression, but there were no significant differences between them. Instead of this, selection ratios were strongly correlated with personality traits estimated from a model’s neutral face in single-person presentation situations. These results suggested that the factors on which participants place importance in judgments during confrontation situations depend on how long they can consider them.

When participants rated personality while viewing a single face, ratings were strongly correlated between extremely short exposure durations (i.e., 100 ms) and unconstrained exposure durations (Willis and Todorov, 2006). Willis and Todorov’s results indicated that first impressions are important in face evaluations. However, the results of this study were inconsistent with those findings. That is, in confrontation scenes, people with happy expressions were regarded as good, trustworthy, and attractive in shorter exposure durations, but not in longer exposure durations. In single-person situations, observers had to infer models’ personalities from limited cues, whereas, in confrontation scenes, they could compare individuals with different identities in longer exposure durations. Therefore, participants may infer personality traits and use them in longer exposure durations. In everyday life, we sometimes interact with only one person, while at other times, we interact with multiple individuals for relatively longer time (e.g., classroom, lecture, group interview, or cocktail party). We are likely to choose useful personal evaluation factors depending on group size and the amount of interaction time.

Even in confrontation situations, dominance perception does not change depending on exposure durations (Ueda and Yoshikawa, 2018). The results in this study suggest that judgments concerning the trustworthiness dimension (the first principal component of trait judgment) and dominance dimension (the second principal component of trait judgment) have different underlying rules even though both judgments were made in confrontation situations. Perceived dominance, i.e., which of two people is more dominant, comprises judgments on the current situation or relationships between individuals (i.e., situationally dependent; see also Ueda et al., 2017). Since facial expressions precisely demonstrate the current status of individuals, dominance judgments rely strongly on this information. However, perceived trustworthiness and attractiveness comprise judgments on individuals’ traits rather than their relative status. Thus, although facial expressions affect instantaneous judgment, people may determine who to be with/trustworthiness/attractiveness based on more information than just facial expressions, especially when they can view faces for a longer time in confrontation situations.

In our series of experiments, we also manipulated the intensity of facial expressions. Our results demonstrated the same tendency regardless of intensity, suggesting that it was unlikely that participants did not interpret facial expressions into different personalities in accordance with their intensity in judgments of confrontation situations. Furthermore, each participant was presented with only the male–male pairs or the female–female pairs. Then, although there were some differences in which personality traits were accounted for when making deliberative judgments, the main pattern of results (which facial expression was chosen more) was almost consistent regardless of model sex and participant sex. Happy expressions were the most chosen, but in some combinations of model sex and participant sex, the differences between happy and neutral expressions were slightly smaller. Note that these results were exploratory and did not aim to examine the effects of these combinations; therefore, they should be further investigated in the future.

It is known that happy expressions have higher arousal than other expressions (e.g., Mancini et al., 2020). In this study, the recognizability was matched across expressions, and it can be considered that the higher this rating, the higher the level of arousal. Therefore, the results of this study would not be affected by arousal. It should be noted, however, that arousal was not directly measured. Moreover, when examining the effects of facial expressions, like in this study, seven facial expressions (neutral, happiness, anger, disgust, fear, sadness, and surprise) were often used (e.g., Ueda and Yoshikawa, 2018). However, this study used only four (neutral, happiness, anger, and sadness) because the number of facial expressions had to be reduced to include temporal manipulations and ensure a sufficient number of repetitions per condition. Since these four facial expressions included both positive and negative valences and are representative of facial expression research, it is unlikely that the use of fewer expressions would have produced the results of this study.

Recent studies have shown that emotional facial expressions elicit consistent, replicable behavioural effects only when they are related to participants’ goals and not when other features (e.g., gender) are relevant (Mirabella, 2018; Mancini et al., 2020, 2022; Mirabella et al., 2022). Since these studies have only examined the situations where just single faces were presented and no data for two-person confrontation situations, it would be useful to extend the paradigm of this study to such studies. This study only examined judgments about social features implying emotions or facial expressions, and did not investigate the judgments about non-social features. We would like to examine the influence of facial expressions on these features in the future.

In this study, we focused on confrontation situations and succeeded in providing robust empirical evidence in such situations. In real life, however, our social interaction situations are greatly diverse and are not limited to confrontation interaction scenes. Therefore, in future studies, we should investigate whether the findings observed in confrontation situations can be adapted to a variety of social interaction situations (e.g., more than two persons interacting simultaneously). Furthermore, although the pairs presented in this study looked like peers, we should also investigate decisions on pairs with different relationships (e.g., based on sex, and age). Our previous study (i.e., Ueda and Yoshikawa, 2018) and the current study suggest that perceived relationships between two persons and the choice behaviour rule differ between single-person and confrontation situations. Researchers should question the idea that personality traits inferred in single-person situations also apply in social interaction scenes. Moreover, they should accumulate empirical results concerning what people perceive in actual interaction situations.

## Data availability statement

The datasets presented in this study can be found in online repositories. The names of the repository/repositories and accession number(s) can be found at: https://osf.io/8ymqv/.

## Ethics statement

Ethical review and approval was not required for the study on human participants in accordance with the local legislation and institutional requirements. The participants provided their written informed consent to participate in this study. Written informed consent was obtained from the individual(s) for the publication of any potentially identifiable images or data included in this article.

## Author contributions

YU and SY planned the experiments and contributed to the interpretation of the results. YU carried out the experiments, analyzed the data, and wrote the original draft. All authors contributed to the article and approved the submitted version.

## Funding

This research was supported by a Grant-in-Aid for Scientific Research (18H03506 and 19H00628) and a Grant-in-Aid for Scientific Research on Innovative Areas No. 18H04195 and 20H04577 “Construction of the Face-Body Studies in Transcultural Conditions”.

## Conflict of interest

The authors declare that the research was conducted in the absence of any commercial or financial relationships that could be construed as a potential conflict of interest.

## Publisher’s note

All claims expressed in this article are solely those of the authors and do not necessarily represent those of their affiliated organizations, or those of the publisher, the editors and the reviewers. Any product that may be evaluated in this article, or claim that may be made by its manufacturer, is not guaranteed or endorsed by the publisher.
