# Supplementary material for: The effects of facial expressions on judgments of others when observing two-person confrontation scenes from a third person perspective
Source: Front Psychol. 2022 Sep 27;13:856336. doi: 10.3389/fpsyg.2022.856336 (PMC9552665; doi:10.3389/fpsyg.2022.856336)
Supplement: Supplementary file 1 [file Table_1.docx]

# Supplementary Table S1

Mean of the Grade on the Scale of Experiment 1 by Model and Participant Sexes.

|  |  |  |  |  |  |  |
| --- | --- | --- | --- | --- | --- | --- |
| Presentation Duration | Model Sex | Participant Sex | The Average Grade | | | |
|  |  |  | Happy | Neutral | Sad | Angry |
| 500 ms | Women | Women | 5.97 | 2.05 | -3.39 | -4.64 |
|  |  | Men | 4.95 | 1.39 | -3.20 | -3.14 |
|  | Men | Women | 5.31 | 1.31 | -2.75 | -3.86 |
|  |  | Men | 1.59 | 1.91 | -1.64 | -1.86 |
|  |  |  |  |  |  |  |
| 5 sec. | Women | Women | -0.06 | 0.33 | -0.25 | -0.03 |
|  |  | Men | 0.25 | -0.31 | 0.16 | -0.10 |
|  | Men | Women | 0.31 | 0.67 | -0.94 | -0.03 |
|  |  | Men | 0.23 | -0.48 | 0.16 | 0.10 |
