# Supplementary material for: The effects of facial expressions on judgments of others when observing two-person confrontation scenes from a third person perspective
Source: Front Psychol. 2022 Sep 27;13:856336. doi: 10.3389/fpsyg.2022.856336 (PMC9552665; doi:10.3389/fpsyg.2022.856336)
Supplement: Supplementary file 2 [file Table_2.docx]

# Supplementary Table S2

Mean of the Grade on the Scale of Experiment 2 by Model and Participant Sexes.

|  |  |  |  |  |  |  |
| --- | --- | --- | --- | --- | --- | --- |
| Presentation Duration | Model Sex | Participant Sex | The Average Grade | | | |
|  |  |  | Happy | Neutral | Sad | Angry |
| 500 ms | Women | Women | 5.60 | 2.13 | -4.25 | -3.48 |
|  |  | Men | 1.70 | 1.90 | -2.40 | -1.20 |
|  | Men | Women | 2.80 | 2.18 | -1.95 | -3.03 |
|  |  | Men | 2.03 | 0.85 | -1.83 | -1.05 |
|  |  |  |  |  |  |  |
| 5 sec. | Women | Women | 0.28 | -0.25 | 0.13 | -0.15 |
|  |  | Men | -0.08 | 0.13 | -0.15 | 0.10 |
|  | Men | Women | -0.08 | 0.53 | -0.43 | -0.03 |
|  |  | Men | 0.60 | -0.33 | -0.03 | -0.25 |
