# Supplementary material for: The effects of facial expressions on judgments of others when observing two-person confrontation scenes from a third person perspective
Source: Front Psychol. 2022 Sep 27;13:856336. doi: 10.3389/fpsyg.2022.856336 (PMC9552665; doi:10.3389/fpsyg.2022.856336)
Supplement: Supplementary file 3 [file Table_3.docx]

# Supplementary Table S3

Mean of the Grade on the Scale of Experiment 3 by Model and Participant Sexes.

|  |  |  |  |  |  |  |
| --- | --- | --- | --- | --- | --- | --- |
| Presentation Duration | Model Sex | Participant Sex | The Average Grade | | | |
|  |  |  | Happy | Neutral | Sad | Angry |
| 500 ms | Women | Women | 5.36 | 0.69 | -3.00 | -3.06 |
|  |  | Men | 4.95 | 1.89 | -3.39 | -3.46 |
|  | Men | Women | 4.10 | 1.53 | -2.65 | -3.08 |
|  |  | Men | 4.35 | 0.58 | -3.03 | -1.90 |
|  |  |  |  |  |  |  |
| 5 sec. | Women | Women | 0.22 | -0.31 | 0.83 | -0.75 |
|  |  | Men | 0.59 | 0.25 | 0.02 | -0.87 |
|  | Men | Women | 0.33 | -0.10 | -0.08 | -0.15 |
|  |  | Men | 0.78 | -0.15 | -0.13 | -0.50 |
